# The foundation for investigating factor XI as a target for inhibition in human cardiovascular disease

**DOI:** 10.1007/s11239-024-02985-0

**Published:** 2024-04-25

**Authors:** Ahmed E. Ali, Richard C. Becker

**Affiliations:** 1 Department of Internal Medicine, Mayo Clinic, Rochester, MN USA; 2 Department of Internal Medicine, College of Medicine, University of Cincinnati, 231 Albert Sabin Way, Cincinnati, OH 45267 USA

**Keywords:** Factor XI, Monoclonal antibodies, Small molecule inhibitors, Hemostasis

## Abstract

Anticoagulant therapy is a mainstay in the management of patients with cardiovascular disease and related conditions characterized by a heightened risk for thrombosis. Acute coronary syndrome, chronic coronary syndrome, ischemic stroke, and atrial fibrillation are the most common. In addition to their proclivity for thrombosis, each of these four conditions is also characterized by local and systemic inflammation, endothelial/endocardial injury and dysfunction, oxidative stress, impaired tissue-level reparative capabilities, and immune dysregulation that plays a critical role in linking molecular events, environmental triggers, and phenotypic expressions. Knowing that cardiovascular disease and thrombosis are complex and dynamic, can the scientific community identify a common pathway or specific point of interface susceptible to pharmacological inhibition or alteration that is likely to be safe and effective? The contact factors of coagulation may represent the proverbial “sweet spot” and are worthy of investigation. The following review provides a summary of the fundamental biochemistry of factor XI, its biological activity in thrombosis, inflammation, and angiogenesis, new targeting drugs, and a pragmatic approach to managing hemostatic requirements in clinical trials and possibly day-to-day patient care in the future.

## Contact activation pathway of coagulation

The contact activation pathway’s relevance for maintaining hemostatic capacity in vivo has been debated as patients deficient in factor (F) XII, PK (pre-kallikrein), or HK (high molecular weight kininogen) do not typically present with a bleeding phenotype, and Factor XI (FXI) deficiency (hemophilia C), is a mild- to- moderate, tissue-specific bleeding disorder. In addition, both FXII and FXI can be “bypassed” by other constituents of the coagulation pathway that can activate “downstream” proteins and produce sufficient thrombin to attain a fibrin clot [1].

In the past several decades, in vitro and animal model studies have suggested that some proteins of the contact pathway, specifically FXI and FXII, contribute to stable clot formation on aggregated platelets [2]. The procoagulant environment contributing to thrombus formation is not solely influenced by activation of the coagulation cascade and platelets but is also affected by the activity of other systems, including inflammatory and fibrinolytic systems that are influenced by FXI and FXII activity. Therefore, a comprehensive understanding of contact pathway proteins is required to guide future drug development and use in thrombosis-associated conditions.

## Factor XI structure and function

Human Factor XI, a 160-kDa serine protease glycoprotein, circulates as a disulfide-linked dimer of two identical 607 amino acid subunits. Each polypeptide consists of a 35-kDa C-terminal light chain containing the trypsin-like catalytic domain, and an N-terminal 45-kDa heavy chain with four ~ 90 amino acid tandem repeats termed apple domains [3]. These apple domains confer binding to other proteins: A1 contains binding sites to HK and thrombin, A2 and A3 to FIX, A3 to the platelet receptor GP1bα and heparin, and A4 to FXII and the other subunit of FXI [4]. FXIa subsequently activates FIX to FIX a by the cleavage of two activation sites [5].

### Evolutionary biology

It is widely thought that a series of gene duplications throughout evolution led to the complete clotting cascade, as seen in humans. Accordingly, clotting factors contain many of the same conserved domains and are closely related [6]. A simple version of the clotting cascade appeared in the earliest vertebrates, and as vertebrates, themselves have become more complex with more intricate and contained cardiovascular systems, the clotting cascade has evolved similarly. While thrombin was the first coagulation factor to emerge before the appearance of the first vertebrates, the contact pathway was the last to evolve, as genes for HK, FXII, and a single paralog of PK/XI are found later in vertebrate development [6].

### Factor XI synthesis, storage, and expression

FXI is expressed mainly in the liver and is regulated by the transcription factor HNF4α, but FXI mRNA has also been detected in platelets, blood mononuclear cells, granulocytes, pancreas, and kidneys as well [7]. Hemophilia C, or FXI deficiency, is a rare bleeding disorder that affects approximately one in one million people. With over 190 reported mutations in the F11 gene [8], most are Cross-Reactive Material – (CRM −) (Type I) with mutations causing low FXI plasma levels. Fewer mutations are CRM + (Type II) with normal FXI levels but reduced coagulant activity mostly due to functional mutations. Historically, it has been difficult to predict bleeding tendency in FXI deficient patients as there has been a poor correlation between bleeding phenotype and plasma FXI levels. This important topic is discussed in detail in the following section.

### Factor XII-mediated factor XI activation

**Fig. 1:**
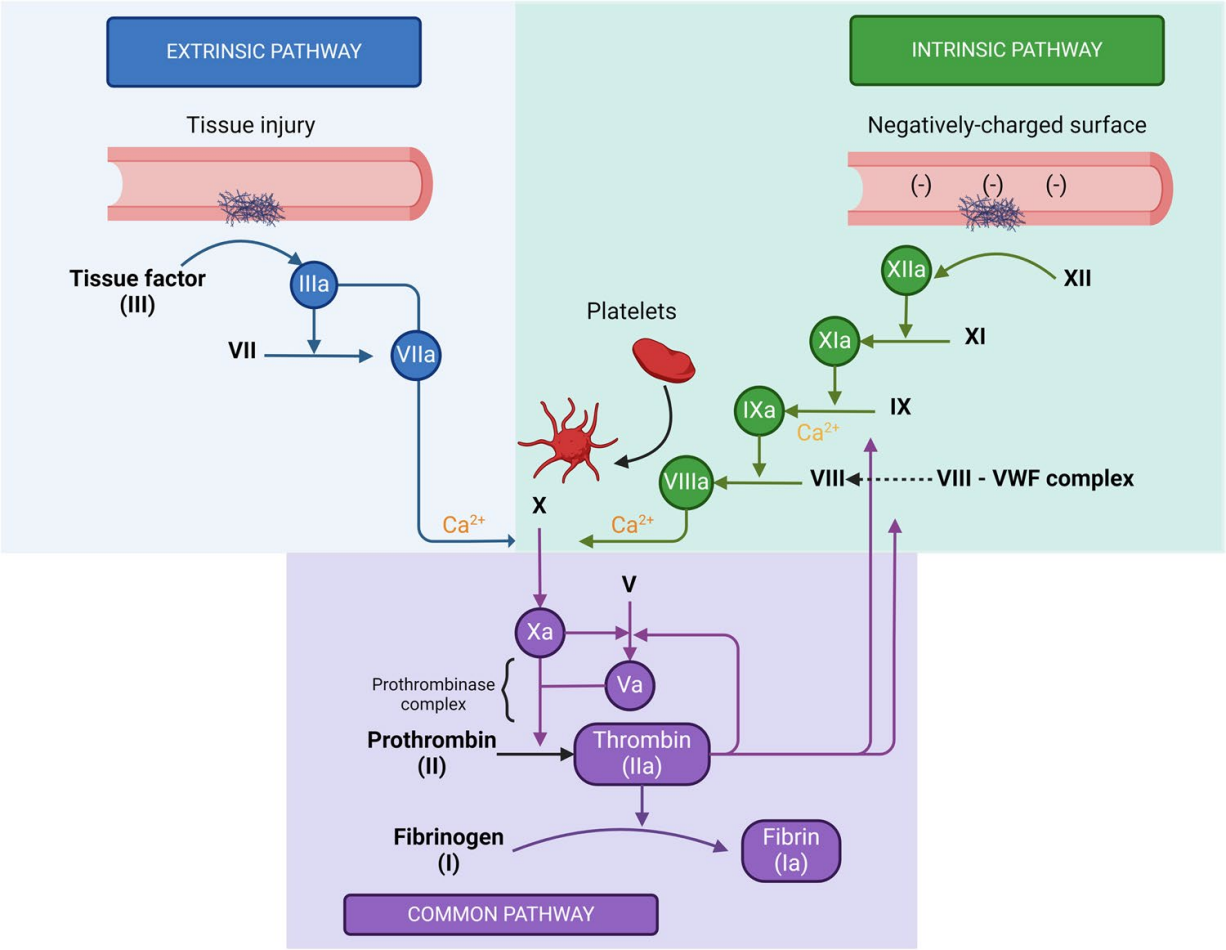
The intrinsic pathway of coagulation also referred to as the contact pathway is initiated following exposure of blood to negatively charged surfaces. The are a series of biochemical/enzymatic steps that follow, including activation of factors XII, XI, IX, and VIII that in complex with factor X (prothrombinase complex), and phospholipid convert prothrombin to thrombin (factor IIa). Thrombin then catalytically converts fibrinogen to fibrin- the predominant structural protein in a thrombus

FXII mediated activation of FXI is a pivotal step in the intrinsic pathway of coagulation, contributing to thrombus formation and hemostasis. Upon exposure to a negatively charged surface, such as activated platelets or subendothelial collagen, FXII undergoes a conformational change leading to its autoactivation to FXIIa (contact activation). FXIIa subsequently cleaves FXI to its activated form, FXIa, initiating a cascade of proteolytic events resulting in fibrin formation (Fig. 1) [9]. Mice lacking FXII or FXI are resistant to experimentally-induced thrombosis, suggesting that FXIIa activation of FXI contributes to thrombus formation in vivo [2]. Interestingly, FXII deficiency does not impair hemostasis, indicating that the FXIIa-mediated FXI activation contributes to thrombus formation while being dispensable for hemostatic processes. This mechanism underscores the significance of FXII-FXI interaction in maintaining hemostatic balance.

### Thrombin-mediated factor XI activation

**Fig. 2:**
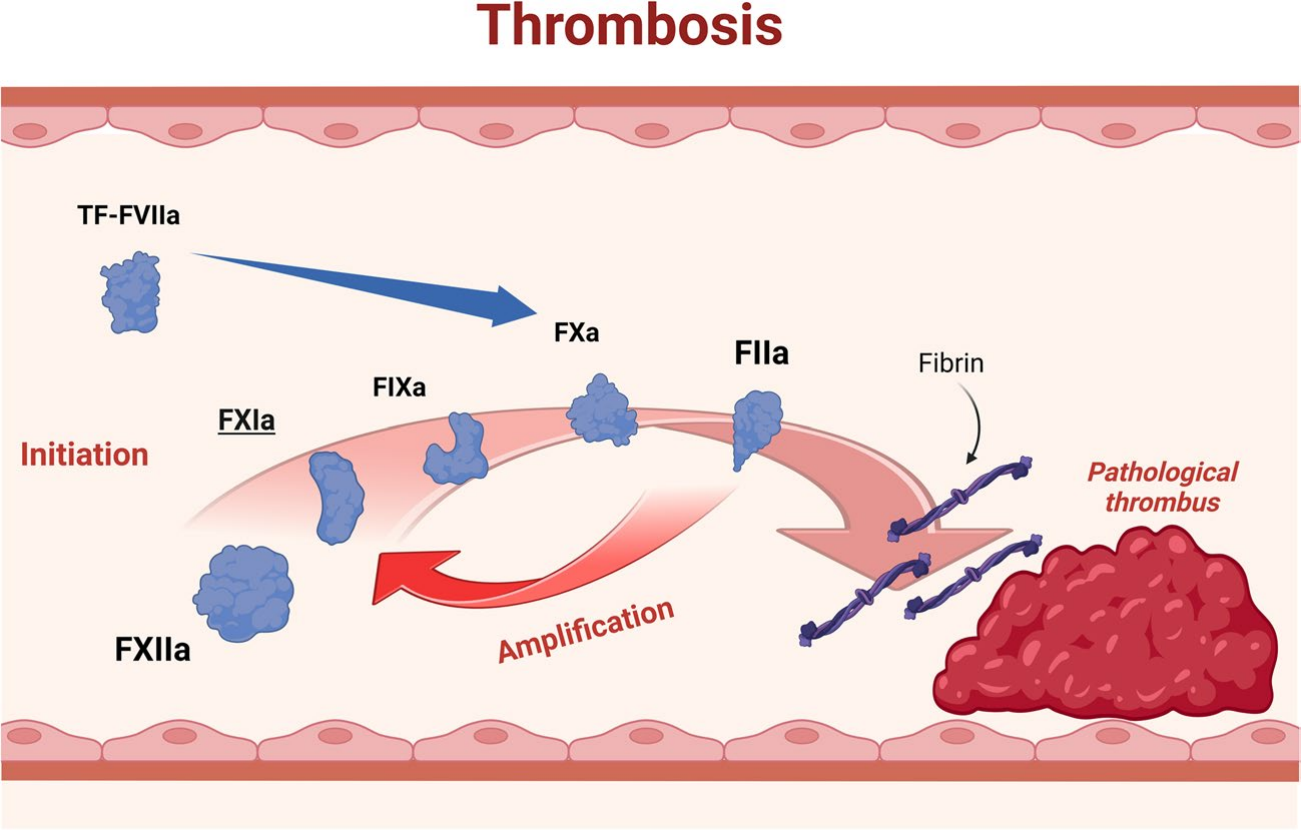
Role of factor XI in thrombosis. Factor XI is particularly important for thrombin generation following activation of the intrinsic pathway of coagulation (see text). “Created with BioRender.com”

The contribution of thrombin-mediated factor XI activation is primarily through the amplification of tissue factor-FVIIa (TF-FVIIa) which participates in both hemostasis and thrombosis (Fig. 2) [10]. The role of factor XI is particularly relevant in low TF organs and tissues, such as the placenta and uterus. The tissue specificity requirement for factor XI aligns biologically with observations made among persons with hemophilia C and other factor XI deficiency states will experience bleeding upon provocation of the same tissues that also possess high fibrinolytic activity including the nose, mouth, and genitourinary tract. These observations also highlight the importance of thrombin activatable fibrinolytic inhibitor (TAFI) [11].

### Factor XI and platelets

Because platelets provide a surface or template for factor XI mediated thrombin activation [12], and platelet-monocyte aggregates as well as micro-vesicles are a source of TF, thrombocytopenia and impaired extracellular vesicle formation contribute to bleeding phenotypes in patients with factor XI deficiency [13]. Thrombin activates FXI at a 5–10,000-fold enhancement when bound to the platelet surface [14]. While there are ~ 1500 binding sites for FXI on the surface of the platelet, FXIa binds to only ~ 250 sites [15], suggesting that FXI and FXIa bind in a different mechanism.

The relationship between factor XI, TF, and platelets is complex, and flow dynamics and shear stress must be taken into consideration to understand the relationship fully [16]. A relatively high concentration of platelets at the site of vascular injury, coupled with subendothelial TF exposure can physically inhibit TF-VIIa activity; this is particularly evident at high shear stress where platelet deposition in a physical boundary to TF complexing with factor FVII occurs more regularly. Proteins present at sites of vascular injury such as C1 inhibitor and platelet-released protease nexin 2 also inhibit fluid phase active factor XI [17].

### Cellular and organ-specific tissue factor activity

TF is selectively expressed in cells and tissues [18], including nonvascular cells [19]. There is a nonuniform distribution, with high levels of TF in the brain, lung, and placenta; intermediate levels in the heart, gastrointestinal tract, kidney, uterus, and testes; and low levels in skeletal muscle, spleen, thymus, and the liver [19].

## Factor XI, molecular biology, and thrombus architecture

Nakazawa and colleagues first identified cell-free (CF) nucleic acids, specifically cfRNA, that initiated coagulation by serving as a cofactor [20]. Kannemeier and colleagues performed a series of experiments to determine the functional significance of intracellular material exposed to blood following tissue injury [21]. Extracellular RNA-activated proteases of the contact system of coagulation, including factor XI itself exhibited strong RNA binding. Extracellular RNA also augmented (auto-) activation of proteases of the contact phase pathway of blood coagulation such as factors XII and XI, both exhibiting strong RNA binding.

Secondary structures of RNA, particularly hairpin-forming oligomers are highly procoagulant. There is an RNA length-contact activation-dependent relationship, however, even relatively short polyphosphates released from activated platelets accelerate factor V activation, inhibit the anticoagulant activity of tissue factor pathway inhibitor (TFPI), promote factor XI activation by thrombin, and are associated with the synthesis of thicker fibrin strands that are resistant in fibrinolysis [22]. Extracellular polyphosphates augment factor XI activation and often co-localize with extracellular DNA and RNA following cellular injury and in inflammatory environments [23].

## Is hemophilia C a disease model for the safety of factor XI inhibitors?

**Fig. 3:**
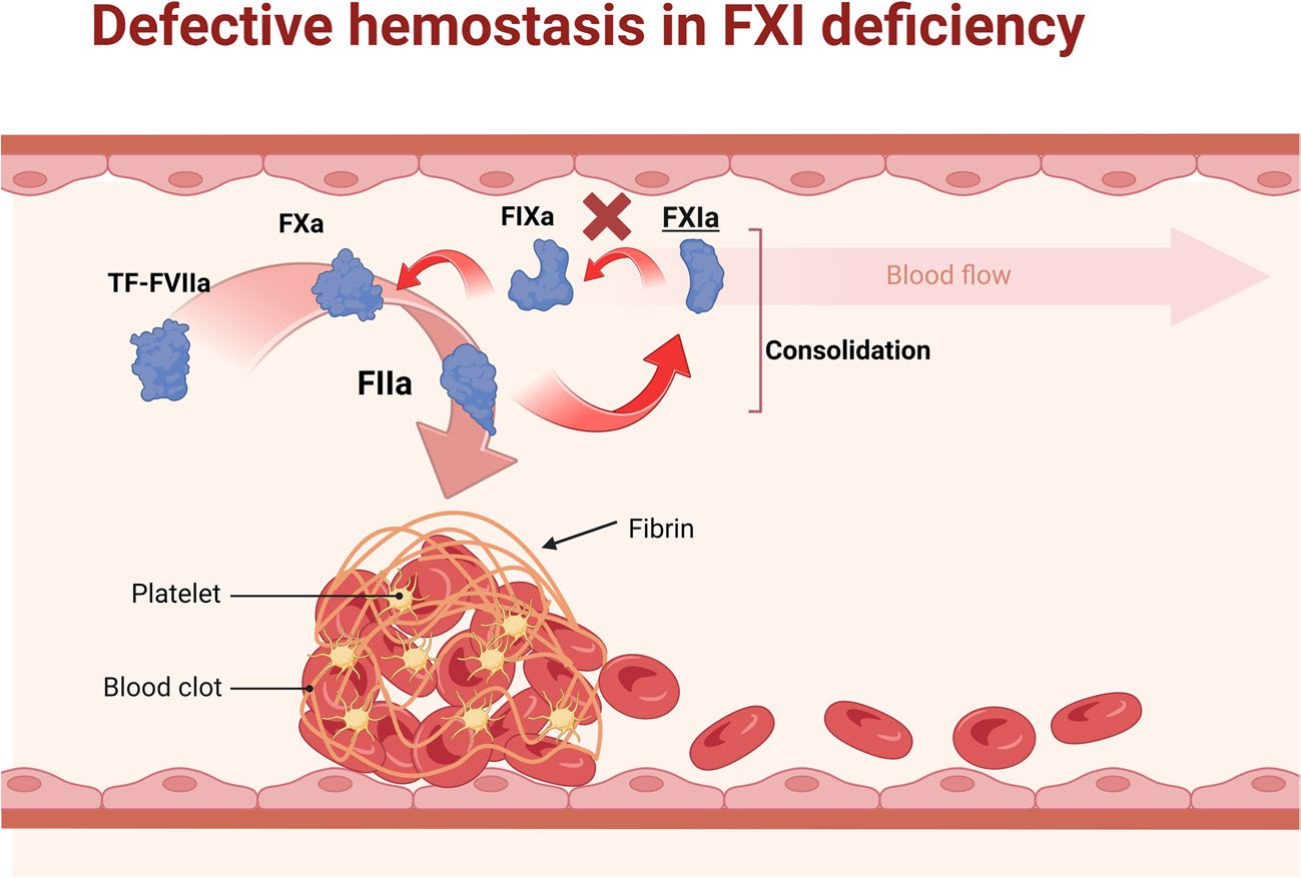
Role of factor XI in hemostasis (see text). Factor XI plays an important role in thrombus consolidation and feedback activation following thrombin activation through the extrinsic or tissue factor pathway of coagulation. “Created with BioRender.com”

Patients with hemophilia C have a deficiency of factor XI. Because bleeding tends to be mild, most often follows a hemostatic challenge, and in most cases does not correlate with factor XI antigen level or activity, complementary roles for other coagulation factors or hemostasis regulating pathways or systems must be considered (Fig. 3). Factor XI can activate coagulation factors X, V, and VIII. It also inhibits tissue factor pathway inhibitor (TFPI) [11].

The activated partial thromboplastin time (APTT) does not correlate with the risk of bleeding in patients with hemophilia C. By contrast, if an APTT is performed with the contact pathway inhibited and using tissue factor (TF) rather than thrombin as an activator, clotting time correlates with a bleeding phenotype [24].

## Hemophilia C and patient-specific factors

The management of patients with hemophilia C provides insights for the optimal selection of patients being considered for treatment with factor XI inhibitors. Guidelines for hemophilia C to include treatment of acute coronary syndrome recommend against coadministration of fibrinolytic therapy [25] and emphasize limiting the duration of dual antiplatelet therapy. Coagulation factor replacement therapy is an option in patients with a bleeding phenotype or further undergoing surgical revascularization. This is discussed in a subsequent section. Whether anti-TFPI antibody should be used in patients with hemophilia C is debated largely because of the infrequent need for replacement products; however, their availability is important and may also play a role in the development of factor XI inhibitors for a variety of clinical indications.

### Hemophilia C and bleeding phenotypes

Investigation of hemophilia C and bleeding phenotypes provides useful information as the area of oral and parenteral factor XI inhibitors begins to take shape. Patients with and those without bleeding tendencies has similar factor XI antigen levels, prothrombin times, fibrinogen, factor XII, von Willebrand factor, and factor XIII concentrations. As previously discussed, clot structure and overall architecture differ among persons with a bleeding phenotype with lower fibrin density and clot stability following decalcification and addition of TF and phospholipids. Moreover, clot stability is highly impaired in the presence of tissue plasminogen activator [26]. In vitro models that combine the measurement of APTT in the rate of clot formation that can be assessed, and fibrinolysis assays may be able to identify patients at particularly high risk for bleeding.

### Tissue Factor pathway inhibitor

Human TFPI is a protein composed of 3 Kunitz-type domains flanked by peptide segments. The K1 domain inhibits FVIIa complexed with TF, while the K2 domain inhibits factor Xa [27]. The TFPI-FXa complex is a potent inhibitor of TF-VII. The K3 domain participates in factor Xa kinetics on the vascular endothelial cell surface. Vascular endothelial cells are the primary site of full-length TFPI synthesis and release both constitutively and in response to vascular injury when hemostasis is required. Platelets also secrete TFPI that resides on the surface of activated platelets [28]. While the kinetics of TFPI, more specifically its clearance, is predominantly regulated by the reticuloendothelial system, vascular endothelial cells are involved with internalization and recycling particularly when TFPI is in complex with factor Xa [29].

Following thrombin generation, endothelial cells synthesize and release TFPI, increasing local anticoagulant effects [30]. Exogenous inhibition of coagulation factors Xa, IXa, and VIIa can increase cell surface TFPI [31], potentially augmenting their anticoagulant effects and intrinsic resistance to thrombus formation [32].

### TFPI and bleeding phenotypes

A bleeding phenotype in factor XI deficiency states is characterized by reduced clot formation, decreased fiber network density, and increased susceptibility to fibrinolysis (Fig. 3). In vitro models of factor XI deficiency have shown that anti-TFPI antibody administration can increase thrombin generation [33]. Because patients with factor XI deficiency are dependent on TF-mediated thrombin generation, TFPI plasma concentration is believed to be important for characterizing and distinguishing patients with either a low or high likelihood of bleeding [34].

### Why might hemophilia C increase TFPI levels?

Elevated TFPI activity has been described in patients with unclassified bleeding disorders [35] and in both hemophilia A and B [36]. By contrast, TFPI is not a major determinant of thrombin generation in healthy individuals [37].

### Do factor XI inhibitors influence TFPI kinetics?

As briefly mentioned in the preceding section, TFPI is an anticoagulant protein that attenuates the initiation phase of coagulation before thrombin is generated [38]. TFPIs action is uniquely positioned to TF-expressing surfaces and sites of vascular injury. In addition, TFPI-α and TFPI-β localize to specific sites and dampen both cellular trafficking and signaling pathways governed by TF-mediated coagulation. A full appreciation of TFPI activity has led to the development of specific inhibitors for the management of hemophilia-associated bleeding [39] as well as bleeding complications that occur in rare bleeding disorders [40].

Because factor XI proteolytically cleaves TFPI, factor XI targeted therapies may increase TFPI thereby increasing overall anticoagulant effects [41]. A pro-fibrinolytic state may also ensue because of reduced TAFI [41]. Further investigation will be required to better understand the impact of factor XI targeted therapies on endogenous fibrinolysis and anticipated effects with short and long-term administration.

## Factor XI and inflammation

Activation of the contact system has been shown to induce inflammatory reactions through activation of the classical cascade of the complement system, leading to anaphylatoxin production causing smooth muscle cell contraction, enhanced vascular permeability, and recruitment of inflammatory cells to produce a local immune response [42]. In addition, contact pathway activation leads to the release of BK, a small peptide that is cleaved from HK by kallikrein, which induces vascular permeability and binds to its receptor on endothelial cells to promote nitric oxide and prostacyclin release, inducing smooth muscle cell relaxation and vasodilation [43]. Constituents of the contact system can also bind to and activate immune cells directly, as kallikrein can induce neutrophil homing, aggregation, and degranulation [43].

Humans with VTE display a proteomics signature that supports an association between factor XI and inflammation. Specifically, factor XI activity is increased and correlates with proteins linked with thrombo-inflammation, oxidative stress, extracellular matrix formation, and apoptosis. Several examples include cell adhesion protein (Spon1), cytokine binding proteins, NF kappa beta, B-cell development, and Th17 cell differentiation [44]. There are similar associations in sepsis that cause systemic inflammation, cytokine release, and complement activation. Factor XI deficiency attenuates the inflammatory response to sepsis in animal models and contact-activated disseminated intravascular coagulation (DIC) [45].

## Factor XI and the cardiovascular system

The liver and heart are known to exhibit one-sided dimensional and bidirectional signaling for physiologic homeostasis [46]. For example, patients with advanced cirrhosis have reduced ventricular diastolic and systolic performance. A condition known as cirrhotic cardiomyopathy has been proposed. In murine models, factor XI activates the bone morphogenic protein (BMP)- SMAD 1/5 pathway in the heart and protects against heart failure with preserved ejection fraction [47]. Increased expression of factor XI also reduces cardiac inflammation and fibrosis. Specifically, mice overexpressing factor XI have reduced macrophages, T cells, monocytes, and granulocytes in models of heart failure with preserved ejection fraction.

Inflammation is prevalent in the early stages of venous thrombosis, contributing to vascular remodeling, valve performance, and thrombus propagation. In animal models of VTE, factor XI inhibition is associated with endothelial cell monocyte and macrophage accumulation [48]. This is an area of ongoing investigation.

### Factor XI and atherogenesis

Epidemiology-based observations suggest that factor XI deficiency is associated with reduced incidence of VTE, ischemic stroke, and myocardial infarction [49, 50]. There are data that also support a protective effect on atherogenesis, the proximate substrate for arterial thrombotic events [51]. Ngo and colleagues employed an LDL-R knockout, high-fat mouse model to test defective factor XI inhibition on atherogenesis. The identified reduced atherosclerotic lesion areas in the proximal aorta and aortic sinus with factor XI inhibition achieved with an antisense oligonucleotide for factor XI when compared to untreated controls. In an established atherosclerosis model, they also showed that factor XI inhibition reduced atherosclerotic lesion area and endothelial cell lipoprotein permeability [51].

Ganor et al. reported that Apoprotein E/FXI double knockout mice had a 32% reduction in atherosclerotic lesion area at 24 weeks compared with Apolipoprotein knockout mice [52].

### Factor XI and thrombogenesis

While mice deficient in FXI do not exhibit abnormal bleeding, these mice are protected against thrombus formation in several models of arterial and venous thrombosis, as well as stroke. In several models of vascular injury, intravital microscopy and blood flow monitoring suggest that, while thrombus initiation is not interrupted on the vessel wall in FXI deficient mice, thrombi that are formed are unstable and break up before occlusion of a blood vessel can occur [53]. Protection from thrombosis is not limited to arterial injury, as FXI deficiency also confers a thrombo-protective effect in a transient middle cerebral artery occlusion model (MCAO), with a lower volume of infarcted brain tissue and less fibrin deposition in distal micro-vessels without hemorrhage [54].

## Factor XI inhibitors in development

Based on the differential contribution of FXI in hemostasis and thrombus formation, particularly its interactions in the contact pathway, inhibition of its action has emerged and attracted attention as promising option for anticoagulation therapy. These inhibitors offer a unique therapeutic approach for preventing thrombotic events while potentially reducing the risk of bleeding complications compared to traditional anticoagulants. By specifically targeting Factor XI, these novel agents aim to interrupt the clotting process without affecting other factors essential for hemostasis. Several drugs have been developed or under-developing with different mechanisms. A summary of FXI inhibitors classes and drugs is shown in (Table 1) [55–75]. The general pharmacological characteristics of each class of factor XI inhibitors are summarized in (Table 2). These drugs have been tested in several conditions like total knee arthroplasty (TKA), end-stage renal disease (ESRD), atrial fibrillation (AF), acute ischemic stroke and acute myocardial infarction with promising efficacy and safety outcomes [55, 57–59, 62, 63, 76–79].

**Table 1:** Factor XI inhibitors in development according to class

Class	Drugs
Antisense oligonucleotide drugs	• IONIS-FXIRx/FXI-ASO (ISIS 416858) [54] • Fesomersen (BAY2976217) [55]
Monoclonal antibody drugs	• Osocimab (BAY1213790) [56] • Abelacimab (MAA868) [57] • Xisomab 3G3(AB023) [58] • BAY 1831865 [59] • REGN9933 • MK-2060 • A-336 • aXIMab [60]
Small molecule oral drugs	• Milvexian (JNJ 70033093) (BMS 986177) [61] • Asundexian (BAY 2433334) [62] • ONO-7648 [63] • SHR-2285 [64] • BMS-962212 [65] • EP-7041 (frunexian) [66] • BMS 986209 • ONO-5450598 • Sulfated pentagalloylglucosides [67]
Natural Peptides	• Ir-CPI [68] • Fasxiator [69] • Boophilin [70] • Desmolaris [71] • acaNAP10 [72]
Aptamers	• FELIAP [73] • Aptamer 12.7 [74] • Aptamer 11.16 [74]

**Table 2:** Pharmacological characteristics of factor XI inhibitors

	ASO drugs	Small molecule drugs	Monoclonal antibody drugs	Natural peptides	Aptamers
Mechanism	Block the synthesis of FXI by reducing FXI messenger RNA	Direct inhibition of FXIa though binding to target proteins	Bind to the catalytic domain of FXI and blocks its activation	Bind to specific target proteins thus, block FXI or FXIa activation	Bind to specific target proteins thus, block FXI or FXIa activation
Administration route	SC	Oral or IV	IV or SC	IV	IV or SC
Half-Life (frequency)	Long (weekly to monthly)	Short (Daily)	Long (Monthly)	Short (Daily)	Short (Daily)
Action	Slow and Long acting	Rapid and short acting	Rapid and Long acting	Rapid and short acting	Rapid and short acting
Renal excretion	No	Yes	No	N/A	No
Cyt. P 450 metabolism	No	Yes	No	No	No
Drug-drug interactions	no	Yes	No	Unknown	NO

## Prevention and management of bleeding

The experience in persons with hemophilia C suggests that bleeding events will be uncommon with factor XI inhibitors, however having a plan of management in place is wise should the need arise [80]. This is particularly important following major trauma, surgical procedures involving the genitourinary tract, and when there is a concomitant use of one or more platelet antagonists or fibrinolytic agents (Table 3).

**Table 3:** A summary of possible strategies to prevent bleeding caused by factor XI inhibitors

	Monoclonal antibodies	Small molecule inhibitors	Anti-sense oligonucleotides
Reversal	Could be achieved with monoclonal antibody configured to the target drug (not currently available)	Not available	Not available
Removal	Possible with plasmapheresis (limited data)	Not achievable with dialysis	Not available
Replacement	Early replacement with factor XI concentrate or FFP could saturate unbound drug	Not effective	Replacement therapy with repeated dosing in early stage
Restoration	Likely the most effective approach – could be achieved by tranexamic acid and agents that bypass FXI such as rFVIIa or aPCC	Most effective approach – could be achieved by aPCC, rFVIIa, and antifibrinolytic agents	- Could be achieved by factor XI concentrate and concomitant antifibrinolytic therapy - Potential role for Emicizumab

*FFP* fresh frozen plasma, *rFVIIa* recombinant FVIIa, *aPCC* activated prothrombin complex concentrate

### Pharmacokinetic considerations

The optimal management must take the drug pharmacokinetics into consideration. Specifically, the circulating half-life of the drug and if applicable, the pharmacodynamically active major metabolite, site of metabolism, target (factor XI or XIa), target-drug dissociation constant, and circulating protein binding capacity. For example, a drug with a long circulating half-life that is non-protein bound (i.e., high free plasma concentration) would require a strategy of either *reversal* (of the drug), *removal* from the circulation, or *replenishing* thrombin generation using serial administration of replacement products that are sufficient to restore hemostasis, while at the same time minimizing the risk of thrombosis. By contrast, a factor XI inhibitor with a short circulating half-life that is highly protein-bound may only require a one-time administration of factor XI containing blood replacement products. As always, the severity of bleeding will be the primary determinant of management which could include close observation and local measures to support hemostasis for mild bleeding.

#### General approach

The initial step in the management of bleeding in patients receiving anticoagulant therapy, including a factor XI inhibitor is a careful assessment of clinical status, the need for supportive measures, and the site(s) of bleeding. Volume resuscitation may be required to normalize the blood pressure. Local control of a superficial source, including compression at vascular puncture sites may be sufficient in many cases, while non-compressible sites of bleeding may require systemic therapy or surgical interventions.

#### Intervention strategies

A pragmatic approach to the management of bleeding in patients receiving factor XI inhibitors has four fundamental tenets: *reversal, removal*, *replacement*, and *restoration*.

### Reversal

The reversal of a drug inherently suggests that a specific antidote or decoy binding agent is available- ideally, co-developed with an anticoagulant with the express intent to have a drug-antidote pair or reversal agent for urgent or emergent circumstances that immediately require normal hemostasis. It is important to be aware that rapid reversal in the circulation will prompt the release of drug present within tissue stores (considered as a component of the volume of distribution) that have pharmacodynamic activity. The half-life or administration strategy of the reversal agent, serial bolus, or continuous infusion must be considered and designed accordingly.

Drug-antidote pairs as a proactive development strategy for anticoagulants is encouraged in the academic and private sectors. We believe that even optimal targets for inhibition still may increase bleeding risk-particularly when hemostasis is challenged by trauma, surgery, and use in combination with other antithrombotic agents. Developing a reversal agent *after* an anticoagulant has been approved for clinical use is costly and time consuming. Determining that a reversal agent is needed to address bleeding or mitigate bleeding in high-risk settings can also introduce uncertainty among clinicians and patients.

### Removal

The removal of a drug from the circulation is inherently attractive when severe or life-threatening bleeding occurs or a surgical procedure associated with high-to-very high bleeding risk must be undertaken (e.g., neurosurgery). The most widely available strategy, hemodialysis, will not be a successful option for plasma protein-bound drugs or large molecules. The same may be true for plasmapheresis for protein-bound drugs, but large molecules can typically be removed.

### Replacement

Intravenous infusion of clotting factors is designed to replenish normal plasma concentrations. It may also saturate a drugs binding sites thereby permitting endogenous clotting factors to participate in hemostasis [81].

### Restoration

Restoration in the context of bleeding applies to thrombin generation and clot stability. Providing blood products that are known to generate thrombin by one or more mechanisms may be sufficient to stem bleeding, particularly in areas that are not readily accessible to manual compression or other local measures. This approach is also of benefit and frequently used in combination with an intervention to stop bleeding (e.g., cautery for a gastrointestinal source of bleeding). There are many determinants of clot stability, however, in the context of anticoagulant drugs in general and FXI inhibitors particularly, as well as years of experience with mitigating bleeding risk and managing bleeding among persons with hemophilia C *attenuating endogenous fibrinolysis* and allowing *fibrin generated by thrombin’s activation of fibrinogen* are the primary goals (see sections on recombinant factor VII, concizumab, and antifibrinolytic agents below).

#### Specific approach

##### Monoclonal antibodies

There are specific approaches to bleeding that are determined by the factor XI inhibitor and its associated mechanism of action. Factor XI replacement in the form of factor XI concentrate or fresh frozen plasma (FFP) is a preferred approach with the goal of saturating the drug thereby allowing unbound factor XI to participate in hemostasis. During treatment, factor XI should be measured to avoid very high free plasma concentrations and the potential risk of thrombosis. Factor XI replacement would not be an effective strategy for the management of osocimab-associated bleeding, given its mechanism of action that reduces factor XII-mediated factor XI activation.

Extraction of large molecules such as monoclonal antibodies is a familiar technique to the pharmaceutical industry for manufacturing purposes. These techniques could, at least theoretically, be used to remove a factor XI targeting monoclonal antibody. Extraction using reverse micelles (RM) of an anionic surfactant and a combination of anionic (AOT) and nonionic surfactants is possible [82]. The process, specifically for use on a case-by-case basis in the management of patients with life-threatening hemorrhage after receiving a factor XI monoclonal antibody, requires further investigation.

##### Small molecule inhibitors

Active site inhibitors that target factor XIa and not impacted by replacement products because factor XI itself is not reduced during drug therapy. Optimal management would require either reversal or removal of the drug; however, the currently available small molecules are not dialyzable, and monoclonal antibody reversal agents have not been included in their development. Restoration of thrombin generation using activated prothrombin complex concentrate, recombinant factor VIIa, and antifibrinolytic agents should be considered and administered according to the severity of bleeding.

##### Anti-sense oligonucleotides

This class of drug targets mRNA and inhibits the synthesis of factor XI. It is highly bound to plasma proteins with limited renal clearance and a very long duration of effect. Accordingly, a strategy to attenuate its effects would require long-term replacement. In acute settings, factor XI concentrate would be the optimal approach likely with concomitant antifibrinolytic therapy with a goal to immediately gain control over bleeding.

##### Factor XI concentrates

Factor XI concentrates are plasma-derived but contain a more consistent amount of factor XI when compared with fresh frozen plasma [83]. Recovery of factor XI from plasma is excellent, and the half-life is 40 h or more. The half-life of endogenously, hepatic-synthesized factor XI is 52 h.

There are virally inactivated factor XI concentrates available for clinical use outside of the United States. Both products also contain several other factors to minimize the procoagulant effects of administration such as Antithrombin, C1 esterase inhibitor, and heparin. The anticipated response is a 2% increase in factor XI for every unit per kilogram of factor XI concentrate. Accordingly, 15 units/kg is expected to increase the factor XI level to 30% or more. In many instances, this will be sufficient to restore hemostasis. Repeat dosing to increase factor XI to 50% or more should be considered if bleeding persists or recurs [84, 85].

##### Fresh frozen plasma

Fresh frozen plasma (FFP) is the fluid portion of a unit of whole blood frozen in a designated time frame, usually within 8 h. FFP contains all coagulation factors except platelets. Fresh frozen plasma contains fibrinogen (400 to 900 mg/unit), albumin, protein C, protein S, antithrombin, and TFPI. It is free of erythrocytes and leukocytes. FFP corrects coagulopathy by replacing or supplying plasma proteins in patients who are deficient in or have defective plasma proteins. A standard dose of 10 to 20 mL/kg (4 to 6 units in adults) will raise factor levels by approximately 20%. A rise of approximately 10% in various factors is sufficient to achieve hemostasis. Also, FFP provides some volume resuscitation, as each unit contains approximately 250 ml [86, 87].

There is approximately 1 unit of factor XI per mL of plasma. Accordingly, FFP given at a dose of 10 to 20 mL/kilogram should increase the factor XI level to 10–20% above baseline. The overall volume of fluid administered during replacement with FFP should always be taken into consideration in patients with a history of heart failure.

##### Activated prothrombin complex concentrate

Prothrombin complex concentrate (PCC) comes from the process of ion-exchange chromatography from the cryoprecipitate supernatant of large plasma pools and after the removal of antithrombin and factor XI. Processing techniques involving ion exchangers allow to produce either three-factor (i.e., factors II, IX, and X) or four-factor (i.e., factors II, VII, IX, and X) PCC.

While the absence of FXI from APCC makes it a less attractive alternative for the management of factor XI inhibitor-associated bleed, thrombin generation is still achievable.

A dose of 50 units/kg, with an additional 25 units/kg for uncontrolled bleeding is used in a variety of clinical settings, most often for warfarin-associated bleeding with an elevated INR. Its use in hemophilia has progressively lessened given the presence of alternatives that carry a lower risk of thrombosis [88].

##### Recombinant factor VII

Administration of pharmacologic doses of exogenous rFVIIa enhances thrombin generation on the platelet surface at the site of injury, independently of the presence of FVIII or FIX. Pharmacologic doses of rFVIIa induce hemostasis not only in hemophilia patients, but also in patients with thrombocytopenia, functional platelet defects, and bleeding triggered by surgery or trauma [89].

When administered at pharmacologic doses, rFVIIa binds to the surface of activated platelets in a TF-independent manner and promotes factor X activation, and thrombin generation on the activated platelet surface [90]. The binding of factor VIIa to platelets involves the glycoprotein Ib/IX/V complex and anionic phospholipids expressed on activated platelets.

In non-hemophilic conditions, platelet-bound rFVIIa increases the activation of both factor IX and FX and increases thrombin generation above normal levels [91]. Increased thrombin generation then promotes increased activation and local accumulation of platelets improving hemostasis.

A dose of rVIIa of 15 to 30 mcg/kg may be sufficient to restore hemostasis in patients with mild to moderate bleeding. Repeat dosing every 4–6 h should be considered for uncontrolled bleeding [92]. Higher doses may be needed in the treatment of intracranial hemorrhage (70–90 mcg/kg). It is important to recognize that factor VII has a very short half-life (~ 6 h or less).

### Other considerations

#### Concizumab

Concizumab is a prophylactic therapy for patients with hemophilia. After subcutaneous administration, it binds to the Kunitz-2 binding domain of TFPI and prevents its binding to factor X. In turn, sufficient FXa can be produced by the TF-VIIa complex and participate in hemostasis [93]. The FDA has not approved concizumab in the United States, requesting more information on dosing and monitoring from the manufacturer. There are limited data on the treatment of hemophilia C and no data for the treatment of FXI inhibitors.

#### Emicizumab

Emicizumab is a humanized, specific monoclonal antibody that binds to factors IX and X, mediating factor X activation thereby bypassing the normal mechanism of FVIII-mediated activation that is missing in patients with hemophilia A [94, 95]. The drug is known to affect the accuracy of several laboratory tests that are used in coagulation. These include the activated partial thromboplastin time, activated clotting time, and all APTT-based assays such as FVIII activity and activated protein C (APC) resistance test. Several laboratory tests are not affected by the drug. These include prothrombin time, thrombin time, immune-based assays, and chromogenic assays other than FVIII. Concomitant administration of emicizumab and activated prothrombin complex concentrate is associated with thromboembolic events and thrombotic microangiopathy if the cumulative amount of the latter blood replacement product is greater than 100 units/kg/ 24 h [96]. There are limited data on the treatment of hemophilia C and no data for the treatment of FXI inhibitors.

## Antifibrinolytic agents

The fibrinolytic system is designed to limit the amount of fibrin that is formed following vascular injury by converting plasminogen to plasmin [97]. Poorly regulated thrombus formation would have devastating consequences on tissue perfusion. By contrast, the management of hemophilias to include hemophilia C employ antifibrinolytic agents to generate fibrin, maintain vascular integrity and augment hemostasis. Several agents are widely available at relatively low cost and can be administered by varied routes depending on the site(s) of bleeding or the anticipated risk of bleeding from an invasive procedure. Clinicians should be aware of these agents and their use if factor XI inhibitors become part of the therapeutic armamentarium of anticoagulants in daily practice.

### Tranexamic acid

Tranexamic acid is a synthetic analogue of lysine. It binds to lysine receptor sites on plasminogen blocking its conversion to the active enzyme plasmin and preventing fibrin degradation. It is more potent than epsilon aminocaproic acid. The available formulations and doses are summarized below:

Oral: 650 mg tablets in the United States and 500 mg in other countries

- 1 g 4 times daily
- Mouthwash 5% for dental procedures
- Intravenous for life-threatening hemorrhage—10 mg/kg every 6–8 h.

### Epsilon aminocaproic acid

Epsilon aminocaproic acid, like tranexamic acid, is a lysine analogue that binds competitively to plasminogen, preventing its binding to fibrin, generation of plasmin, and the degradation of fibrin. The available formulations and doses are summarized below:

- Oral: 5 to 6 g daily
- Oral rinse: 15 mL solution containing 1.25 g/5 mL over 2 min.

## Conclusion

Comprehensive understanding of the systems impacted by the contact pathway is crucial, as it influences numerous cardiovascular disorders. While FXI is thought to have a limited role in hemostasis where it is primarily activated to FXIa by thrombin, its role in thrombosis can vary depending on the event. When the extrinsic pathway is inhibited, the contact pathway plays a significant role in thrombus formation; however, FXI may not be essential for thrombosis at a ruptured plaque where a large amount of tissue factor can trigger independent thrombin generation. Moreover, FXI has additional participation in inflammation, angiogenesis, and fibrinolysis. As a result, the contact pathway, involving factor XI (FXI), has attracted great attention as a potential target for safer anticoagulants developments. Different classes of FXI inhibitors have been developed including antisense oligonucleotides, monoclonal antibodies, small synthetic molecules, natural peptides, and aptamers with different mechanisms. The clinical data has shown a promising effect of FXI inhibitors in preventing thrombosis without a concomitant increase in the bleeding burden. Additional large-scale clinical trials are needed to confirm the safety of these drugs on the long-term and support their use in different medical scenarios.

## Data Availability

All data supporting the findings of this review are available within the paper and cited references.
